# Increased risk of thyroid cancer in female residents nearby nuclear power plants in Korea: was it due to detection bias?

**DOI:** 10.1186/s40557-018-0233-0

**Published:** 2018-04-10

**Authors:** Bong-Kyu Kim, Jung-Min Kim, Myoung-Hee Kim, Do-Myung Paek, Seung-Sik Hwang, Mi-Na Ha, Young-Su Ju

**Affiliations:** 1 Department of Occupational & Environmental Medicine, Hallym University Sacred Heart Hospital, Anyang, South Korea; 2 Department of Environmental Health Sciences, Graduate School of Public Health, Seoul National University, Seoul, South Korea; 3 Department of Occupational & Environmental Medicine, Cheong Ju Medical Center, Cheongju, Korea; 4 Department of Medical Science, Graduate school of Hallym University, Chuncheon, South Korea; 5 The People’s Health Institute, Center for Health Equity Research, Seoul, South Korea; 6 Department of Public Health Science, Graduate School of Public Health, Seoul National University, Seoul, South Korea; 7 Department of Preventive Medicine, Medical College, Dankook University, Yongin, South Korea

**Keywords:** Nuclear power plant, KREEC, Thyroid cancer, Radiation, Detection bias

## Abstract

**Background:**

The Korea Radiation Effect & Epidemiology Cohort - The resident cohort (KREEC-R) study concluded that there is no epidemiological or causal evidence supporting any increase in cancer risks resulting from radiation from Korean nuclear power plants (NPPs). But the risks of thyroid cancer in women were significantly higher in residents living near NPPs than control. Debate about the cause of the pattern of thyroid cancer incidence in women is ongoing and some researchers argue that detection bias influenced the result of KREEC-R study. Therefore there was a need to investigate whether residents living near NPPs who were assessed in the KREEC-R were actually tested more often for thyroid cancer. We evaluated the possibility of detection bias in the finding of the KREEC-R study based on materials available at this time.

**Methods:**

Using the KREEC-R raw data, we calculated age standardized rates (ASRs) of female thyroid cancer and re-analyzed the results of survey on the use of medical services. We also marked the administrative districts of residents who received the Radiation Health Research Institute (RHRI) health examinations and those in which thyroid cancer case occurred as per the Chonnam National University Research Institute of Medical Sciences (RIMS) final report on maps where the locations of NPPs and 5 km-radii around them were also indicated. And we compared the incidence rates of Radiation-induced cancer measured between the first period when RHRI health examinations were not yet implemented, and the second period when the RHRI health examinations were implemented.

**Results:**

The ASR for the far-distance group, which comprised residents living in areas outside the 30 km radius of the NPPs, increased rapidly after 2000; however, that of the exposed group, which comprised residents living within a 5 km radius of the NPPs, started to increase rapidly even before 1995. The frequencies of the use of medical services were significantly higher in the intermediate proximate group, which comprised residents living within a 5–30 km radius of the NPPs, than in the exposed group in women. In case of female thyroid cancer, the second period ASR was higher than the first period ASR, but in case of female liver cancer and female stomach cancer no significant difference were observed between the periods. On map, many administrative districts of residents who received RHRI health examinations and most administrative districts in which thyroid cancer case occurred on RIMS final report were outside 5 km-radii around NPPs.

**Conclusions:**

We could not find any evidence supporting the assertion that detection bias influenced the increased risks of female thyroid cancer observed in the exposed group of the KREEC-R study, as opposed to the control group.

## Background

KREEC-R study was a prospective cohort study aiming to evaluate cancer risks in residents living near four Korean NPPs, and in NPP employees. The study was conducted between December 1991 and February 2011. Based on a total of 303,524 person-years of follow-up data, the KREEC study group concluded that there is no epidemiological or causal evidence supporting any increase in cancer risks resulting from radiation from NPPs [1, 2].

However, when each cancer type associated with radiation was assessed, the risks of thyroid cancer in women were significantly higher in residents living near NPPs than in those living in other areas.

Some researchers argued that these results were found because residents living near NPPs were screened more often for thyroid cancer due to the health examination benefits provided by NPPs and local governments. This raises the possibility that the KREEC-R finding regarding thyroid cancer risks in women was influenced by detection bias [3].

Detection bias, which is a form of selection bias, refers to bias in the correlation between disease-causing factors and diseases when subjects with risk factors are screened more often than others [4].

In fact, the incidence of thyroid cancer in Korea has rapidly increased since 2000, reportedly due to increases in screening [5]. Therefore, there was a need to investigate whether residents living near (within a 5 km radius) NPPs who were assessed in the KREEC-R were actually tested more often for thyroid cancer.

As such, due to the need to consider detection bias in the findings of the KREEC-R study and the need to re-analyze the overall data of the study, re-analysis of the KREEC-R data began in 2013, and the final report was submitted in 2015 [6]. The aims of the re-analysis study were as follows: 1) to replicate the findings of the KREEC-R study based on the KREEC-R data; 2) to evaluate the influence of reviewable factors on the risks of major cancers, including thyroid cancer, by conducting a case-control study within the cohort; and 3) to evaluate the possibility of detection bias in evaluating the risks of thyroid cancer in women, in light of the issues raised by some researchers.

The present manuscript aims to evaluate the possibility of detection bias in the finding of the KREEC-R study based on materials available at this time.

## Methods

### Previous study [1]

The exposed cohort in the KREEC-R was defined as residents living within a 5 km radius of four Korean NPPs (Yeonggwang, Kori, Uljin, and Wolsung). The non-exposed cohort was divided into the intermediate proximate group (control-1 group), which comprised residents living within a 5–30 km radius of the NPPs (control-1 study area), and the far-distance group (control− 2 group), which comprised residents living in areas outside the 30 km radius of the NPPs (control-2 study area). The cohort of KREEC-R study was open cohort and the enrollment had been conducted from 1992 to 2006. And question investigation on experience of medical service use in the KREEC-R study was done at the point of enrollment for each participant. The total follow-up duration was 303,542.5 person-years (PY). The follow-up began in 1992 and was cut-off in 2008.

### Data sources

1) We received the raw data and the final report of KREEC-R from the Korea Foundation of Nuclear Safety (KoFONS), which commissioned the re-analysis study.

2) The Radiation Health Research Institute (RHRI), which is an organization affiliated with the Korea Hydro & Nuclear Power Co., Ltd. (KHNP) that is responsible for operating all Korean NPPs, conducted health examinations of 7728 residents living in administrative districts near the four Korean NPPs between 2004 and 2011. In health examinations conducted between 2004 and 2008, thyroid tests, such as thyroid ultrasonography, were included. Therefore, there was a need to investigate whether the RHRI health examinations were indeed provided more often to residents residing within a 5 km radius of the NPPs. For this, we reviewed the RHRI Medical Service Result Report [7].

3) The Chonnam National University Research Institute of Medical Sciences (RIMS) conducted a study between 1997 and 1999 to investigate why the incidence of thyroid cancer was significantly higher in Yeonggwang-gun than in other areas; the final report for this study was submitted to RHRI in 2006. In order to examine whether the 91 Yeonggwang-gun residents with thyroid cancer, as shown in the final report, resided within a 5 km radius of Yeonggwang NPP, we reviewed the RIMS final report [8].

### Geographic and statistical analysis

Using the KREEC-R raw data, we calculated ASR of female thyroid cancer in the exposed, control-1, and control-2 groups of the four NPPs.

Moreover, since the KREEC-R raw data included the results of surveys conducted on the use of medical services by residents belonging to each group, this set of data was re-analyzed using ordered logistic regression.

However, we excluded 150 subjects who were eliminated from the cohort for unknown reasons at unknown time points.

We also marked the administrative districts of residents who received RHRI health examinations between 2004, the year in which the RHRI health examinations began, and 2008, the year in which the KREEC-R follow-up ended, on maps where the locations of NPPs and 5 km-radii around them were also indicated. Through this, we qualitatively analyzed the degree to which the administrative districts of residents who received RHRI health examinations overlapped with the exposed areas, as defined by the KREEC-R study.

Furthermore, for a more quantitative analysis, we compared the incidence rates of thyroid cancer measured between 1992 and 2003, when the RHRI health examinations were not yet implemented, and between 2004 and 2008, based on the KREEC-R data. Since tests for stomach and liver cancers, such as upper gastrointestinal series and abdominal ultrasonography, were included in the RHRI health examination starting in 2006, the incidence rates of stomach and liver cancers measured between 1992 and 2005, and between 2006 and 2008 were compared. If the increase in female thyroid cancer risks observed in the exposed group is indeed due to increased RHRI health examinations, then the incidence of thyroid cancer in the exposed group would have significantly increased during the years in which the RHRI health examinations were conducted. Similarly, the incidence rates of stomach and liver cancers would also have increased since 2006. However, since screening tests for breast cancer, which is also associated with radiation, were not included in the RHRI health examinations, no analysis of breast cancer was conducted.

For comparison of incidence rates, ASRs for cancer incidence during each time period, for each sex, and for each region were calculated based on Segi’s world standard population [9]. Respective 95% confidence intervals (CI) were also calculated.

Through its final report submitted to RHRI in 2006, RIMS concluded that the incidence of thyroid cancer in Yeonggwang-gun significantly increased between 1997 and 1999 when compared to that of other regions; they suggested that this was because of frequent thyroid cancer tests conducted at two internal medicine clinics in Yeonggwang-gun. Between 1997 and 2003, thyroid cancer was detected in 91 patients included in the RIMS final report; this coincided with the follow-up duration of KREEC-R.

Therefore, there was a need to investigate whether the 91 thyroid cancer patients resided in the exposed areas, as defined by the KREEC-R study.

For this, the administrative districts in which thyroid cancer patients included in the RIMS final report lived were marked on maps. The locations of NPPs and 5 km-radii around the NPPs were also indicated on these maps. This enabled us to qualitatively investigate how the administrative districts overlapped with the exposed areas, as defined by the KREEC-R.

All statistical analyses were conducted using Stata version 13.1 (StataCorp., College Station, TX, USA).

## Results

### Female thyroid Cancer incidence rates – Based on raw data of KREEC-R study

Table 1 illustrates Numbers and Follow-up durations of sub-cohort group in KREEC-R study. Tables 1 and 2 illustrates cancer incidence rate per 100,000 person-years by sub-cohort group. (Table 2) In males, the ASR of all cancer cases was 566.2 in the exposed cohort and 545.2 in the non-exposed cohort. In females, the ASR of all cancer cases was 307.0 in the exposed cohort and 281.2 in the non-exposed cohort. For thyroid cancer in female the ASR was 60.7 in the exposed cohort and 33.0 in the non-exposed cohort.

**Table 1:** Numbers and Follow-up durations of sub-cohort group in KREEC-R study^a^

Region	Sex	Numbers	Follow-up period (person-year)
Exposed	Male	4470	43,455.4
	Female	6839	57,658.7
Control-1	Male	4424	40,158.9
	Female	5860	48,487.6
Control-2	Male	6040	45,904.3
	Female	8393	67,674.3
Total	Male	14,934	129,518.6
	Female	21,092	173,820.6

^a^Analysis based on raw data from Korea Foundation of Nuclear Safety [2]

**Table 2:** Cancer incidence rate per 100,000 person-years by sub-cohort group^a^

Cancer	Region	CR(Male/Female)	ASR(Male/Female)
All	Exposed	904.4/541.1	566.2/307.0
	Control	1093.4/560.4	545.2/281.2
	Control-1	1048.3/618.7	509.9/306.5
	Control-2	1132.8/518.7	567.9/264.0
RI cancer	Exposed	572.9/308.3	358.6/189.3
	Control	667.7/313.2	343.1/161.2
	Control-1	614.4/352.2	295.8/181.5
	Control-2	714.3/285.3	375.0/146.5
Stomach ca.	Exposed	229.2/104.4	140.7/49.5
	Control	218.7/109.0	104.0/51.1
	Control-1	191.4/130.3	96.0/58.8
	Control-2	242.5/93.7	110.3/44.5
Liver ca.	Exposed	151.4/22.2	97.6/9.5
	Control	149.4/43.3	75.6/17.7
	Control-1	129.5/32.5	64.9/13.3
	Control-2	166.8/51.5	85.6/21.1
Lung ca.	Exposed	151.4/34.1	90.9/13.2
	Control	256.7/59.5	105.9/22.5
	Control-1	251.7/69.0	107.1/26.6
	Control-2	261.1/52.6	104.7/19.9
Thyroid ca.	Exposed	22.6/75.3	15.9/60.7
	Control	15.9/48.5	11.4/33.0
	Control-1	26.8/61.0	16.3/43.1
	Control-2	6.4/39.5	8.2/26.4
Breast ca.	Exposed	−/56.5	−/44.7
	Control	−/38.3	−/29.7
	Control-1	−/40.6	−/30.3
	Control-2	−/36.6	−/29.0

CR: Crude Rate per 100,000 person-years, ASR: Age-Standardized Rate per 100,000 person-years,

All: all sites, RI cancer: Radio-inducible cancer

^a^Analysis based on raw data from Korea Foundation of Nuclear Safety[2]

**Fig. 1:**
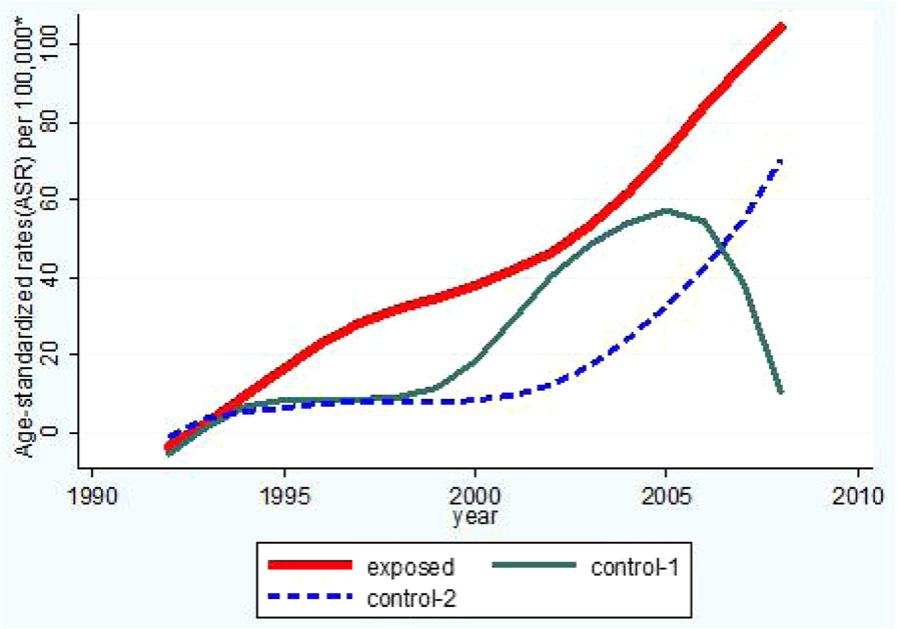
Comparison of thyroid cancer incidence rates by sub-cohort group(Female). *Approximated by Lowess smoothing

Using the raw data of the KREEC-R study, we calculated the ASR of female thyroid cancer in the exposed, control-1, and control-2 groups for each year and analyzed tendencies using the Lowess method. The ASR for the control-2 group increased rapidly after 2000; however, that of the exposed group started to increase rapidly even before 1995. The ASR of the control-1 group increased rapidly from the late 1990s; however, it decreased from the mid-2000s (Fig. 1).

### Comparison of medical service use – Based on raw data of KREEC-R study

We re-analyzed the results of survey on the use of medical services, which were included as raw data in the KREEC-R study, using ordered logistic regression; we found that the frequencies of radiologic tests, including X-ray, upper gastrointestinal series, and CT scan, were significantly higher in the control-1 group than in the exposed group in women. However, in men, there was no statistically significant difference between the three groups (Table 3).

**Table 3:** Experience of X-ray, upper gastrointestinal series, CT scan^a^

Gender	Experience of Radiologic test	Exposed(%)	Control-1(%)	OR^b^	95% CI	Control-2(%)	OR^b^	95% CI
Male	No experience	905(21.5)	746(18.0)	1.04	0.96–1.13	593(12.2)	0.96	0.89–1.05
	< 2	303(7.2)	312(7.5)			514(10.5)		
	22–10	2068(49.1)	2112(51.0)			2736(56.0)		
	1 > 10	940(22.3)	974(23.5)			1039(21.3)		
	Total	4216(100.0)	4144(100.0)			4882(100.0)		
Female	No experience	936(14.5)	558(10.3)	1.35	1.26–1.46	1015(15.7)	0.92	0.86–0.99
	< 2	957(14.9)	664(12.3)			959(14.8)		
	-2-10	3999(62.1))	3618(66.8)			3850(59.5)		
	1 > 10	545(8.5)	574(10.6)			650(10.0)		
	Total	6437(100.0)	5414(100.0)			6474(100.0)		

^a^Analysis based on raw data from Korea Foundation of Nuclear Safety [2], acquired data at the point of enrollment for each participant, 1992–2006

^b^Odds Ratio: ordered logistic regression, adjusted by education and marriage

### Comparison of administrative districts in which RHRI health examinations were implemented and exposed areas

**Fig. 2:**
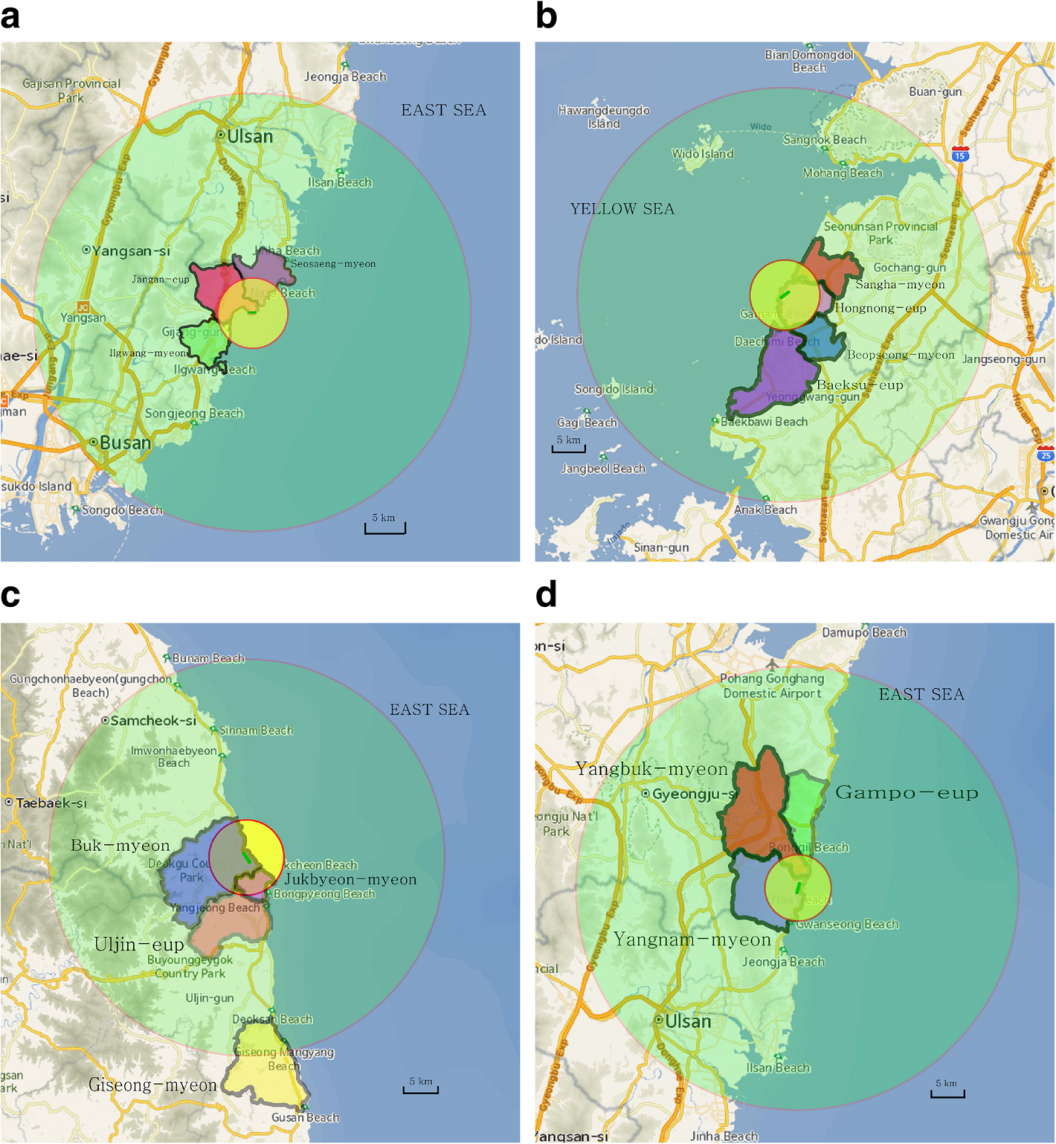
Administrative districts in which Radiation Health Institute provided medical examinations and circles with a radius of five kilometers from NPPs*. **a** Administrative districts nearby Kori NPP: Seosaeng-myeon, Ulju-gun (violet area), Ilgwang-myeon, Gijang-gun (green area), Jangan-eup, Gijang-gun (pink area). **b** Administrative districts nearby Yeonggwang NPP: Baeksu-eup, Yeonggwang-gun (violet area), Beopseong-myeon, Yeonggwang-gun (blue area), Hongnong-eup, Yeonggwang-gun (pink area), Sangha-myeon, Gochang (red area). **c** Administrative districts nearby Uljin NPP: Uljin-eup, Uljin-gun (red area), Giseong-myeon, Uljin-gun (yellow area), Buk-myeon, Uljin-gun (blue area), Jukbyeon-myeon, Uljin-gun (violet area). **d** Administrative districts nearby Wolsung NPP: Yangbuk-myeon, Gyeongju-si (red area), Yangnam-myeon, Gyeongju-si (violet area), Gampo-eup, Gyeongju-si (green area). Black lines: boundary lines of administrative districts, yellow circles with red boundary lines: areas within a radius of five kilometers from NPPs, green circles with red boundary line: areas within a radius of 30 kilometers from NPPs(There is no administrative district outside a radius of 30 kilometers from NPPs except Giseong-myeon.), green rounded rectangular: boundary lines of NPP sites. *Each center of circle is set at each center of NPP site. All maps adapted from National Geographic Information Institute, South Korea. http://map.ngii.go.kr. Accessed 14 Jun, 2017

When the administrative districts of residents receiving health examinations were marked on maps, together with the locations of NPPs and 5 km-radii around the NPPs, administrative districts with RHRI health examination include all area within a radius of 5 km from NPPs (Fig. 2). But overall, administrative districts with RHRI health examination contains more areas corresponding to the control-1 study areas than the exposed areas. Only about 19% of the total area of administrative district covered by RHRI’s health examination was included in the exposed area (Table 4). Thus, the argument that the exposed group would have more opportunities to receive RHRI health examinations than control-1 group is not reasonable.

**Table 4:** The area of the administrative districts near the NPP that conducted RHRI health screenings^a^

NPP	①Total area of administrative district nearby NPP with RHRI examination	②Area within a radius of 5 km from NPP(②/① ratio, %)	③Area within a radius of 30 km from NPP (③/① ratio, %)
Kori	123.64 km^2^	32.00 km^2^ (25.88%)	123.64 km^2^ (100.00%)
Yeounggwang	191.43 km^2^	36.33 km^2^ (18.98%)	191.43 km^2^ (100.00%)
Uljin	340.54 km^2^	62.42 km^2^ (18.33%)	263.43 km^2^ (77.36%)
Wolsung	249.95 km^2^	41.19 km^2^ (16.48%)	249.95 km^2^ (100.00%)
Total	905.56 km^2^	171.94 km^2^ (18.99%)	828.45 km^2^ (91.48%)

^a^Data from Radiation Health Research Institute [8]

### Comparison of radiation-induced Cancer incidence rates

#### Thyroid cancer

When the incidences of thyroid cancer between 1992 and 2003 (ASR-T1), and between 2004 and 2008 (ASR-T2) were compared, ASR-T2 was higher than ASR-T1 in the exposed, control-1, and control-2 groups in women. For men, ASR-T2 was higher than ASR-T1 in the control-1 group; however, in the exposed and control-2 groups, the 95% CIs of ASR-T1 and ASR-T2 were overlapped (Table 5).

**Table 5:** Comparison of Radio-inducible cancer incidence rates by period and sub-cohort group^a^

cancer	Region	Sex	Year	CR	ASR(95% CI)
Thyroid ca.	Exposed	Male	1992–2003	7.9	4.7(0–11.2)
			2004–2008	41.8	26.7(7.5–45.9)
		Female	1992–2003	35.3	32.0(11.8–52.3)
			2004–2008	112.9	83.0(53.6–112.4)
	Control-1	Male	1992–2003	4.6	2.9(0–8.4)
			2004–2008	52.1	29.8(10.2–49.5)
		Female	1992–2003	35.7	13.5(4.0–23.1)
			2004–2008	82.2	73.0(34.6–111.4)
	Control-2	Male	1992–2003	4.7	7.3(0–21.5)
			2004–2008	7.8	8.9(0–21.7)
		Female	1992–2003	19.5	10.2(0.9–19.6)
			2004–2008	55.8	39.9(21.1–58.7)
Liver ca.	Exposed	Male	1992–2005	144.8	104.6(74.4–134.8)
			2006–2008	169.7	85.9(46.1–125.7)
		Female	1992–2005	17.7	8.5(1.9–15.2)
			2006–2008	31.5	9.6(1.0–18.2)
	Control-1	Male	1992–2005	122.4	68.0(45.0–91.0)
			2006–2008	147.8	55.2(26.1–84.4)
		Female	1992–2005	33.3	14.2(5.2–23.2)
			2006–2008	30.7	9.8(0.4–19.2)
	Control-2	Male	1992–2005	171.3	89.1(62.1–116.1)
			2006–2008	158.2	80.7(45.4–116.0)
		Female	1992–2005	46.8	20.1(10.8–29.4)
			2006–2008	59.4	21.6(9.0–34.3)
Stomach ca.	Exposed	Male	1992–2005	213.2	155.4(117.7–193.1)
			2006–2008	273.6	108.5(69.1–147.9)
		Female	1992–2005	91.3	47.7(30.6–64.7)
			2006–2008	131.6	54.6(28.5–80.7)
	Control-1	Male	1992–2005	211.3	117.2(86.7–147.8)
			2006–2008	140.2	54.3(26.1–82.5)
		Female	1992–2005	130.6	61.4(38.8–84.1)
			2006–2008	129.7	48.1(23.5–72.6)
	Control-2	Male	1992–2005	229.9	98.8(74.8–122.9)
			2006–2008	267.2	129.3(83.7–174.9)
		Female	1992–2005	87.1	45.6(29.2–62.0)
			2006–2008	106.5	41.4(23.0–59.9)

CR: Crude Rate per 100,000 person-years

ASR: Age-Standardized Rate per 100,000 person-years

^a^Analysis based on raw data from Korea Foundation of Nuclear Safety [2]

#### Liver cancer

When the incidences of liver cancer between 1992 and 2005 (ASR-L1), and between 2006 and 2008 (ASR-L2) were compared, the 95% CIs of ASR-L1 and ASR-L2 were overlapped in any of the three groups (Table 5).

#### Stomach cancer

Similarly, the incidences of stomach cancer between 1992 and 2005 (ASR-S1), and between 2006 and 2008 (ASR-S2) were compared. ASR-S2 was lower than ASR-S1 in the male control-1 group. However, for men, the 95% CIs of ASR-S1 and ASR-S2 were overlapped in the exposed and control-2 groups. For women, the 95% CIs of ASR-S1 and ASR-S2 were overlapped in any of the three groups (Table 5).

### Comparison of administrative districts of Yeonggwang-gun in which thyroid cancer case occurred in 1997–2003 and exposed areas

**Fig. 3:**
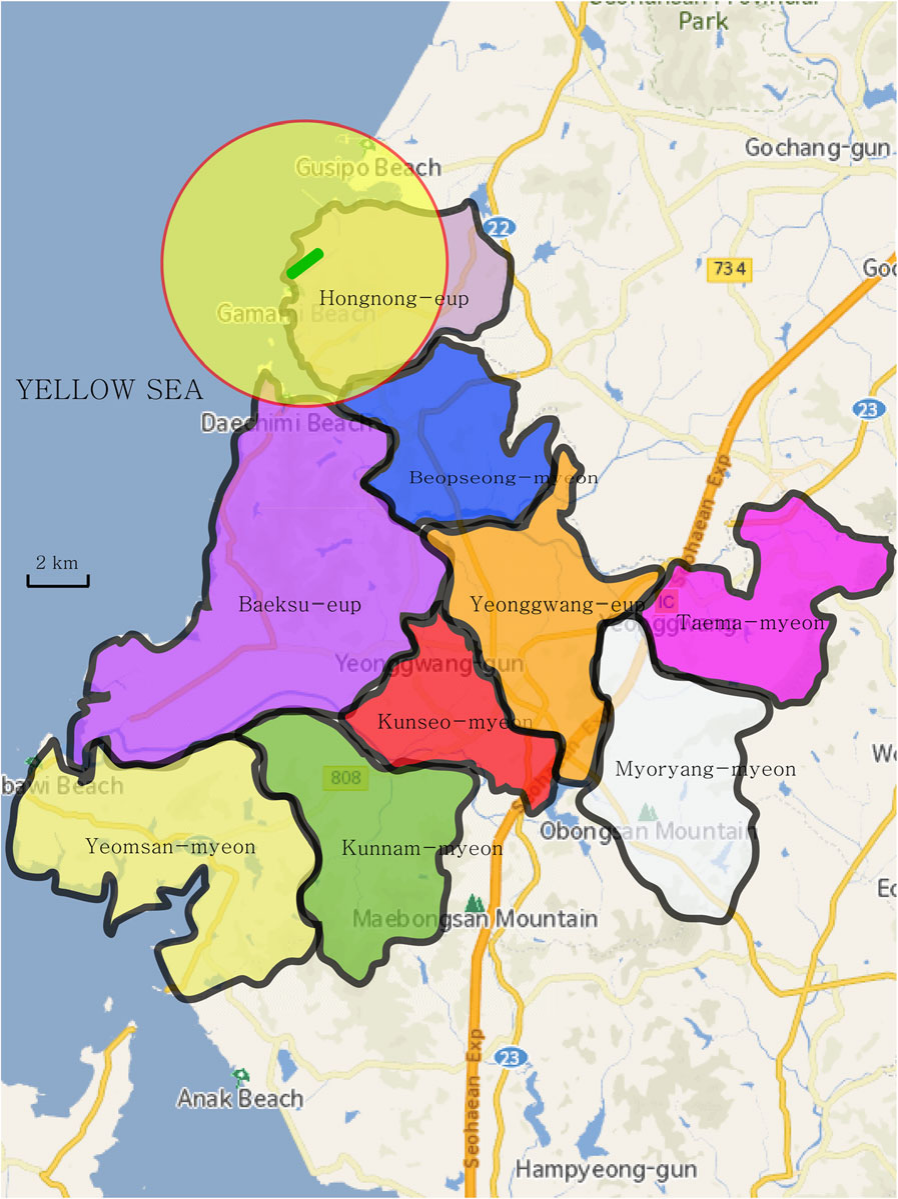
Administrative districts of Yeonggwang-gun in which cases of thyroid cancer occur in 1997–2003 and circles with a radius of five kilometers from Yeonggwang NPPs*: Baeksu-eup (violet area), Beopseong-myeon (blue area), Hongnong-eup (gray area), Kunseo-myeon (red area), Yeonggwang-eup (yellow area), Kunnam-myeon (green area), Yeomsan-myeon (ivory area), Taema-myeon (pink area), Myoryang-myeon (white area). *Black lines: boundary lines of administrative districts, yellow circle with red boundary lines: area within a radius of five kilometers from NPPs, green rounded rectangular: NPP site. *The center of circle is set at center of Yeonggwang NPP site. All maps adapted from National Geographic Information Institute, South Korea. http://map.ngii.go.kr. Accessed 14 Jun, 2017

A total of nine administrative districts of Yeonggwang-gun in which residents were found to have thyroid cancer, as per the final RIMS report submitted to RHRI in 2006, were marked on maps, together with the locations of NPPs and 5 km-radii around the NPPs. This showed that six districts did not overlap at all with the exposed areas. Among the other three administrative districts, only very small parts of two districts (Beopseong-myeon and Baeksu-eup) overlapped with the exposed areas. Only in Hongnong-eup did most of the district correspond to the exposed area (Fig. 3). No administrative district was located outside the 30 km-radius of the NPPs, and therefore, these were not marked on the maps.

Based on the data used in the RIMS report and the map images shown in Fig. 3, we estimated the ratio of exposed area residents among thyroid cancer patients from Yeonggwang-gun who were diagnosed between 1997 and 2003. When only those residing in Hongnong-eup, most of which was classed as an exposed area, were counted as the exposed group, 12 out of a total of 91 patients (13.2%) were in the exposed group. Seventy-nine (86.8%) were classified as the control-1 group (Table 6).

**Table 6:** Number of thyroid cancer patients diagnosed in 1997–2003 by regions^a^

Region	Number of thyroid cancer patients diagnosed in 1997–2003	Ratio (%)	Overlap with the exposed area	Ratio (%)	
Kunnam-myeon	7	7.7	No	52.7	86.8
Kunseo-myeon	7	7.7	No		
Taema-myeon	3	3.3	No		
Younggwang-eup	25	27.4	No		
Yeomsan-myeon	3	3.3	No		
Myoryang-myeon	3	3.3	No		
Beopseong-myeon	13	14.3	Extremely small area	47.3	
Baeksu-eup	18	19.8	Extremely small area		
Hongnong-eup	12	13.2	The great majority of area		13.2
Total	91	100		100	100

^a^Data from Korea Foundation of Nuclear Safety [6] and Radiation Health Research Institute [8]

In the same way, the ratio of exposed area residents among female thyroid cancer patients from Yeonggwang-gun diagnosed between 1997 and 2003 was estimated. When only those living in Hongnong-eup, most of which counted as the exposed area, were counted as the exposed group, 10 out of a total of 74 patients (13.5%) were classified as the exposed group, while 64 (86.5%) were in the control-1 group (Table 7).

**Table 7:** Number of female thyroid cancer patients diagnosed in 1997–2003 by regions^a^

Region	Number of thyroid cancer patients diagnosed in 1997–2003	Ratio (%)	Overlap with the exposed area	Ratio (%)	
Kunnam-myeon	6	8.1	No	51.4	86.5
Kunseo-myeon	5	6.8	No		
Taema-myeon	3	4.1	No		
Younggwang-eup	19	25.7	No		
Yeomsan-myeon	2	2.7	No		
Myoryang-myeon	3	4.1	No		
Beopseong-myeon	10	13.5	Extremely small area	48.6	
Baeksu-eup	16	21.7	Extremely small area		
Hongnong-eup	10	13.5	The great majority of area		13.5
Total	74	100		100	100

^a^Analysis based on data from Korea Foundation of Nuclear Safety [6] and Radiation Health Research Institute[8]

## Discussion

Quantitative evaluation of differences in the use of medical services between the exposed and control groups of the KREEC-R study requires access to the medical records of the cohort. However, due to the reinforced privacy protection act, we could not obtain direct access to individual medical records. Moreover, screening tests for thyroid cancer are often conducted in Korea as a part of comprehensive medical testing, and these data are not registered in the Korean National Health Insurance Corporation database [10]. Therefore, even if we had access to individual medical records registered in the National Health Insurance Corporation, it would have been impossible for us to quantitatively confirm whether significant differences were present in the rates of screening tests for thyroid cancer between the exposed and control groups of the KREEC-R study.

Consequently, among the raw data from the KREEC-R study, we re-analyzed the data on female thyroid cancer and the use of medical services. Moreover, we also investigated factors that could have caused significant differences in the rates of screening test for thyroid cancer between the exposed and control groups.

When the data on female thyroid cancer and the use of medical services were re-analyzed, the incidence of thyroid cancer in the exposed group was found to have started to increase rapidly before 2000, the year in which the nationwide incidence of thyroid cancer started to increase due to more screening. Specifically for women, the use of medical services by the control-1 group was higher than that of the exposed group.

Moreover, we also qualitatively assessed how administrative districts in which residents who received RHRI health examinations between 2004 and 2008 resided overlap with the exposed areas defined in the KREEC-R study using map images. From this, we estimated that the RHRI health examinations wouldn’t have been provided more to residents of the exposed areas than control-1 group.

For quantitative analysis, we investigated the incidence rates of thyroid, stomach, and liver cancers measured during the entire cohort follow-up period based on the KREEC-R data; in particular, we compared the rates measured before and after the start of RHRI health examinations.

There is no increase in the incidence rates of stomach and liver cancer after the start of RHRI examinations in all groups. For female thyroid cancer, the incidence rate increased after the start of RHRI examinations in all groups. For male thyroid cancer, an increase in the incidence rate was observed in the control-1 group after the start of RHRI examinations.

Therefore, no evidence seems to support the assertion that RHRI health examinations increased the use of medical services in the exposed group of the KREEC-R study when compared to other groups.

Furthermore, we also marked the administrative districts in which thyroid cancer patients resided, reported in the 2006 RIMS report submitted to RHRI, on maps on which the locations of NPPs and 5 km-radii of the NPPs were also indicated. Out of nine administrative districts, only Hongnong-eup was found to have been an exposed area. Hongnong-eup residents accounted for 13.2% of all thyroid cancer patients from Yeonggwang-gun who were diagnosed between 1997 and 2003; the other 86.8% of patients were found to have resided in the control-1 area.

Therefore, thyroid cancer screening tests, which were conducted at high frequencies at two internal medicine clinics in Yeonggwang-gun during the KREEC-R study follow-up, seem to have been conducted mainly in the control-1 area, rather than in the exposed area. In other words, this cannot explain why women in the exposed group of the KREEC-R study were found to have higher risks of thyroid cancer than those in the control group.

Putting the aforementioned results together, the pattern of risks of female thyroid cancer observed in the exposed group of the KREEC-R study cannot be explained by detection bias. Therefore, investigation of all causes, including the influences of NPPs, would be necessary.

The explanatory hypothesis is that there was a period of high levels of radiation exposure at the beginning of each NPP operation, and because of that exposure the incidence rate of thyroid cancer among women who were vulnerable to radiation exposure increased.

According to ‘bathtub curve’ of reliability engineering, the intensity function of failure of a system gradually decreases from a high value as time changes at start. And after a certain value is maintained for some time, it gradually shows an increasing pattern of change. A stricter definition of bathtub curve is as follows [11]: We can define a point A1, before which the dominant failure mode is so called ‘infant failure’. From the starting time to A1, the intensity function of failure is roughly decreasing. We can also define another point A2, after which the intensity function of failure is increasing due to wear-out of the system. And between A1 and A2, the intensity function of failure is roughly constant. The bathtub curve has been the basic theoretical background for studies to minimize the failure rate of NPP [12, 13].

In reality, failures of or accidents at NPPs mostly occurred soon after the initial operation, as confirmed by data collected by the Korea Institute of Nuclear Safety (KINS) [14]. Moreover, according to the environmental radioactivity comprehensive evaluation report compiled by the Korea Electric Power Corporation (KEPCO), I-131 was detected in seaweed collected from sea water near Kori NPP in 1974 and 1975 at levels of 180.0 pCi/kg-fresh and 165.7 pCi/kg-fresh, respectively [15]. Furthermore, between 1978 and 1980, gross beta activities were detected within the range of 0.2–1.2 pCi/m3 in airborne particulate samples from areas near Kori NPP [16].

Although there is no confirmative evidence which shows that thyroids of women are more vulnerable to radiation exposure than those of men, a pooled analysis of seven studies reported that the pooled excess relative risk per Gy was greater for women than men (*P* = 0.07) [17]. Furthermore it was reported that parity potentiated the radiation-induced risk of thyroid cancer in a case-control study [18]. This is presumed to be due to hormonal changes.

A review of the models used to evaluate radiation near NPPs is also required. In its 2010 report, the European Committee on Radiation Risk (ECRR) pointed out that the use of the dose evaluation model of the International Commission on Radiological Protection (ICRP) for internal exposure can cause “serious misuse” [19].

Moreover, investigation of pathological findings of thyroid cancer detected near NPPs can offer useful information. Multiple studies of this type were performed in post-Chernobyl thyroid cancer patients [20–25]. In particular, Lydia Zablotska et al. (2015) measured individual thyroid radiation doses within 2 months of the accident, and conducted surveys to estimate thyroid I-131 doses in a cohort of 11,664 Belarus residents who were younger than 18 years of age at the time of the Chernobyl NPP accident. Moreover, the authors conducted three sets of thyroid cancer screening tests between 1997 and 2008; in the tests, those suspected of having thyroid cancer underwent fine needle aspiration biopsy. When an operation was deemed necessary, appropriate surgery was conducted, and the collected specimens were used for pathological biopsy. The authors confirmed that higher radiation doses to the thyroid gland are associated with solid and diffuse sclerosing variants of PTCs, more biologically aggressive cancers, and a higher probability of multifocal cancers and multiple nodular pathology [26].

Therefore, by investigating thyroid cancer cases observed in the exposed and control-1 study areas of the KREEC-R study, it will be possible to confirm whether more aggressive pathological findings are observed when patients live nearer NPPs.

### Limitation

Since we did not have direct access to the individual medical records of the KREEC-R study cohort, and since screening tests for thyroid cancer are often conducted as part of private comprehensive medical testing, it was impossible to investigate the rates of screening tests for thyroid cancer in each cohort group.

Thus, in the re-analysis study, we planned to conduct surveys in residents and conducted a pilot investigation; however, recall bias could not be controlled. Therefore, analysis was instead conducted using the limited data available.

## Conclusion

We re-analyzed the raw data from the KREEC-R study; specifically, we assessed the data on female thyroid cancer and the use of medical services. Moreover, among factors that could potentially cause differences in the rate of use of medical services between the exposed and control groups, we investigated factors that can be reviewed at present. We could not find any evidence supporting the assertion that detection bias influenced the increased risks of female thyroid cancer observed in the exposed group of the KREEC-R study, as opposed to the control group.

## Abbreviations

ASR: Age standardized rate
CI: 95% confidence interval
KEPCO: Korea Electric Power Corporation
KHNP: Korea Hydro & Nuclear Power Co., Ltd.
KINS: Korea Institute of Nuclear Safety
KoFONS: Korea Foundation of Nuclear Safety
KREEC-R: The Korea Radiation Effect & Epidemiology Cohort - The resident cohort
NPP: Nuclear power plant
PY: Person-years
RHRI: Radiation Health Research Institute
RIMS: Chonnam National University Research Institute of Medical Sciences
